# Role of circadian transcription factor REV-ERB in cardiovascular diseases: a review

**DOI:** 10.3389/fcvm.2025.1516279

**Published:** 2025-04-04

**Authors:** Chunling Wang, Jiashu Yang, Jianfang Yuan, Xuyong Wang, Qianrong Li, Chunzhen Ren, Xiaodong Zhi, Xinfang Lv, Kai Liu, Xinke Zhao, Yingdong Li

**Affiliations:** ^1^ School of Traditional Chinese and Western Medicine, Gansu University of Chinese Medicine, Lanzhou, Gansu, China; ^2^ Department of Geriatrics, Affiliated Hospital of Gansu University of Chinese Medicine, Lanzhou, Gansu, China; ^3^ Gansu Medical College, Pingliang, Gansu, China

**Keywords:** circadian rhythm, transcription factor, REV-ERB, cardiovascular diseases, biological clock regulation

## Abstract

Circadian rhythm, or the biological clock, is an intrinsic timing system present in organisms that operates on a cycle of approximately 24 h. Nearly every cell in the human body adheres to a specific circadian rhythm, governing various biological processes essential for overall health. REV-ERB, a key circadian clock-regulating gene, plays a crucial role in maintaining the precision of these rhythms. This gene influences many downstream targets associated with diverse pathophysiological processes, including metabolism, autophagy, immunity, inflammation, and aging across multiple organs. REV-ERB specifically impacts cardiac systolic function by regulating myocardial energy metabolism. In contemporary society, health and well-being are increasingly challenged by disruptions to the biological clock, such as night shifts, late-night activities, and jet lag. These disruptions often lead to circadian rhythm disorders, which are now being increasingly linked to heart diseases. This review explored the potential role of REV-ERB in the cardiovascular system. Beyond its role in circadian rhythm regulation, REV-ERB could significantly influence physiological and pathological processes related to cardiovascular health, including atherosclerosis, myocardial ischemia/reperfusion injury, and heart failure. Mechanistically, REV-ERB could regulate glucose and lipid metabolism, inflammation, autophagy, ferroptosis, and mitochondrial function. The review highlighted the protective roles and underlying mechanisms of REV-ERB in cardiovascular diseases, suggesting that multidisciplinary research may provide a basis for breakthroughs in REV-ERB-targeted therapies for cardiovascular disorders.

## 1 Introduction

Circadian rhythms are critical in regulating various physiological cardiovascular functions, e.g*.*, heart rate and blood pressure, demonstrating rhythmic fluctuations. For instance, blood pressure typically peaks in the morning and declines at night (1, 2) Moreover, certain cardiovascular conditions, such as myocardial infarction (MI), arrhythmias, stroke, heart failure (HF), and sudden cardiac death, exhibit a close association with circadian rhythms, with a higher incidence of these events occurring in the early morning hours (3).

The rapid industrialization of society over the past century has significantly altered the human external environment. The clear distinction between day and night has been disrupted by artificial lighting and frequent travel across time zones. Shift work, in particular, has been linked to an increased risk of both non-cardiac and cardiovascular diseases (CVDs), including acute myocardial infarction (AMI). Among patients with acute coronary syndrome, shift work has been associated with a 15% higher risk of major cardiovascular events and worsened long-term cardiac outcomes (4, 5).

The biological clock is composed of a central clock, located in the suprachiasmatic nucleus of the hypothalamus, and peripheral clocks that function in nearly all tissues (6). The central clock receives optical signals from the retina, which synchronizes peripheral clocks through neurohumoral signals (7). In addition to central clock inputs, peripheral clocks respond to tissue-specific cues, such as food intake and physical activity (8–10).

**Figure 1:**
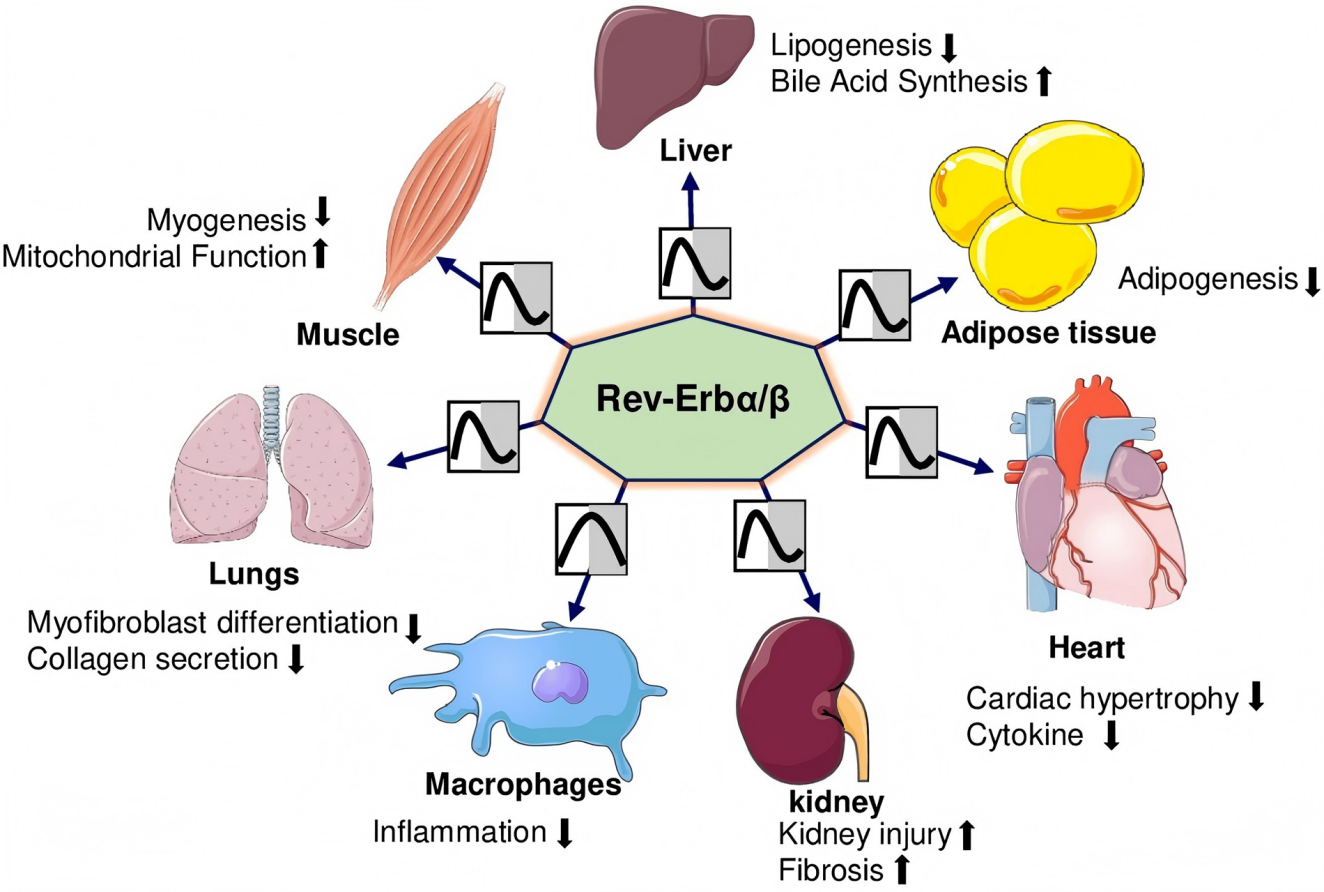
REV-ERBα/βs regulate the organs’ physiological functions. REV-ERBs suppress adipogenesis and lipogenesis while promoting bile acid synthesis in adipose tissue and the liver. In the heart, they mitigate cardiac hypertrophy and reduce inflammatory cytokine levels. In the lungs, they inhibit myofibroblast differentiation and collagen production. In skeletal muscles, REV-ERBs decrease myogenesis while enhancing mitochondrial function. However, in the kidneys, they contribute to increased injury and fibrosis.

The nuclear receptor subfamily 1, group D, member 1 (NR1D1, also known as REV-ERBα) was first identified in 1989 (11). In 1994, researchers discovered a related orphan receptor with high homology to REV-ERBα, named NR1D2 or REV-ERBβ REV-ERBα/β proteins (12), essential components of the circadian clock, are widely expressed across tissues and have become key therapeutic targets for heart diseases (13). REV-ERBs regulate glucose, lipids, energy metabolism, adipogenesis, and inflammation (Figure 1).

This review discusses the role of REV-ERBα/β in circadian rhythm regulation and its association with processes, such as autophagy, inflammation, immunity, metabolism, fibrosis, and ferroptosis. It also examined the links between circadian rhythms and CVDs, emphasizing potential therapeutic strategies. Ligands targeting REV-ERBα/β or retinoic acid-related orphan receptor (RORα/γ), as well as lifestyle modifications, are noticeable for the prevention and treatment of CVDs.

## 2 Circadian rhythm and molecular clock

In 1729, De Maran, a French astronomer, observed human physiological rhythms persisted without external cues, such as light. However, the concept of an intrinsic biological clock remained unaccepted until the 20th century (14). Circadian rhythm refers to behavioral, physiological, and metabolic cycles with a 24-h periodicity, evolved to synchronize biological functions with Earth's rotation. Derived from Latin, meaning “about a day”, this internal timing system ensures optimal adaptation (15). In 2017, Jeffrey C. Hall, Michael Rosbash, and Michael W. Young received the Nobel Prize in Physiology or Medicine for elucidating the molecular mechanisms of the biological clock. Since then, research has underscored its crucial role in human physiology and disease.

The biological clock is a conserved, endogenous timing system across animals, plants, fungi, and bacteria, operating on a 24-h cycle. It regulates physiological processes through rhythmic patterns, which are influenced by genetic, endocrine, behavioral factors, and external cues like light and temperature fluctuations (16). Despite external influences, the circadian system remains self-sustaining (17). Nearly all mammalian cells contain circadian rhythm molecules, with regulation centralized in the hypothalamic suprachiasmatic nucleus (SCN). This pacemaker, composed of ∼15,000 neurons, processes light signals received via the retinal hypothalamic tract, synchronizing peripheral oscillators through hormonal, autonomic, and temperature-mediated pathways (18–20). Strong intercellular coupling within the SCN maintains functional stability and coherence (18).

While the SCN orchestrates the circadian system, peripheral tissues can maintain independent rhythms influenced by cues like feeding schedules (18). Light exposure is essential in regulating circadian rhythms via SCN-mediated synchronization of peripheral clocks, including REV-ERB pathways. Disruptions in light-dark cycles, such as shift work, desynchronize these clocks, contributing to the progress of circadian rhythm disorders and CVDs. REV-ERB regulates clock genes and metabolic pathways, with light exposure modulating its activity through the SCN. Chronodisruption exacerbates conditions, including atherosclerosis, myocardial fibrosis, and heart failure (HF) (21–24). Harmonization of central and peripheral clocks supports stable physiological and behavioral responses (18).

Given the interaction among light exposure, circadian rhythms, and cardiovascular outcomes, targeting REV-ERB may serve as a therapeutic strategy to mitigate the adverse effects of chronodisruption. The pharmacological activation of REV-ERB has exhibited promise in reducing pathological gene expression and improving myocardial infarction and HF preclinical models, further emphasizing its relevance in light-exposure-related circadian dysfunction. Coordinating central and peripheral systems of circadian rhythm is necessary for optimal physiological functioning and maintaining physical and mental health.

### 2.1 Molecular clock

The composition and regulation of circadian rhythms at the molecular level are as follows. The molecular oscillator of the clock system comprises two interlocking negative feedback loops of gene expression. This standard oscillator refers to the transcriptional translation feedback loop (TTFL). Two transcription factors named circadian locomotor output cycles kaput (CLOCK) and brain and muscle ARNT-like 1 (BMAL1) bind to the E-box DNA motif of the circadian clock-controlled genes (CCGs) and then recruit the co-activating proteins CBP/p300 (one group of histone acetyltransferases) along with Thyroid Hormone Receptor-associated protein 3 (THRAP3), steroid receptor coactivator 2 (SRC-2), and some other peptides, which then activate the period (PER1, PER2 and PER3) and cryptochrome (CRY1 and CRY2) genes in the primary feedback loop along with REV-ERBα and REV-ERBβ in the secondary feedback loop (25). The expression products of these genes include PER1, PER2, CRY1, CRY2, and several other peptides, such as casein kinases 1 ε (CK1 ε), which together form a repressor complex. After reaching the threshold activity level, this repressor complex weakens the transcriptional activity of CLOCK and BMAL1. Therefore, the PER and CRY repressor complex levels are reduced, lifting the inhibition of CLOCK and BMAL1 activity. This allows a new 24-h cycle of PER and CRY transcription and translation to begin, maintaining the circadian rhythm. In the secondary feedback loop, CLOCK–BMAL1 heterodimers bind to E-Box promoters and enhancers of REV-ERBα and REV-ERBβ, regulating their temporal expression as negative nuclear orphan receptors. The REV-ERBβ repressor protein competes with the ROR-Peroxisome proliferator-activated receptor gamma coactivator-1 alpha (PGC-1 α) activator/coactivator to bind to the ROR/REV-ERB-response elements (RORE) in BMAL1 and CLOCK. This leads to the recruitment of the nuclear receptor co-repressor 1 (NCoR1)-histone deacetylase 3 (HDAC3) co-repressor complex, inhibiting the transcription of Bmal1 and Clock genes. The transcription and translation feedback loop based on CLOCK-BMAL1 and ROR/RORE interaction operates in opposite phases aligned horizontally, i.e., at a time difference of 12 h. In addition, the phase difference is regulated by clock output regulators, such as the PAR-bZip (rich in proline and acidic amino groups) transcription factor and E4BP4/NFIL3 (E4BP4, also referred to as interleukin-3 nuclear gene NFIL3), the temporal expressions of which are regulated via primary and secondary feedback loops, respectively (26).

Recent studies (27–29) have highlighted the involvement of non-coding RNAs, including microRNAs (miRNAs) and long non-coding RNAs (lncRNAs), in regulating circadian rhythms at the molecular level. Several miRNAs, such as miR-219 and miR-132, have been found to modulate the expression of core clock components, including CLOCK, BMAL1, PER, and CRY, thereby influencing circadian oscillations. Additionally, lncRNAs, such as Neat1 and locked nucleic acid, have been implicated in stabilizing rhythmic gene expression by interacting with chromatin modifiers and transcriptional regulators. These non-coding RNAs contribute to tuning the transcriptional-translational feedback loop well, reinforcing the robustness of circadian rhythms and linking them to metabolic and physiological processes. Their dysregulation has been associated with circadian misalignment and related disorders, including CVDs and metabolic syndromes. Integrating non-coding RNA regulation into the classical TTFL model expands the understanding of circadian control mechanisms and their broader implications for human health.

### 2.2 Molecular regulators of the circadian clock in the heart

Several cardiovascular parameters, such as heart rate, heart rate variability, electrocardiogram (ECG) waveform, and blood pressure, fluctuate pronouncedly every day (30). Approximately 13% of genes and 8% of proteins involved in cardiac growth, metabolism, and molecular signaling follow rhythmic patterns. These fluctuations align with 24-h variations in cardiovascular neurohumoral factors, such as autonomic, sympathetic, and parasympathetic tone, renin-angiotensin-aldosterone system activity, and cortisol levels (31). Clock-regulated genes drive functional oscillations in various cardiac cell types, including endothelial cells, vascular smooth muscle cells, fibroblasts, cardiomyocytes, and cardiac progenitor-like cells (3).

Disruptions to normal circadian rhythms can significantly affect cardiovascular health due to inadequate sleep, shift work, or jet lag. Adverse cardiovascular events, such as myocardial infarction and stroke, occur more frequently in the early morning (6 a.m. to 2 p.m.), with elevated cardiac injury markers like creatine kinase detected in affected patients (32). Experimental studies show that circadian clock disruption in animal models leads to severe CVDs (23). For example, deletion of the circadian clock gene Bmal1 in mice alters cardiac myosin composition, impairs sarcomere structure, and accelerates age-related dilated cardiomyopathy (24).

Cardiomyocyte-specific Bmal1 knockout (CBK) mice exhibit reduced stroke volume and ejection fraction, progressing to early heart failure and premature death. Deleting Bmal1 from myocardial cells disrupts the circadian expression of Na+ and K+ channels, reducing heart rate and increasing arrhythmia risk, which may lead to sudden cardiac death (33, 34). Despite increasing knowledge of the molecular clock's role in cardiovascular physiology and disease, its potential for improving treatment for CVD remains largely untapped.

## 3 Circadian rhythm inhibitor REV-ERB

### 3.1 Structure and function of REV-ERB

**Figure 2:**
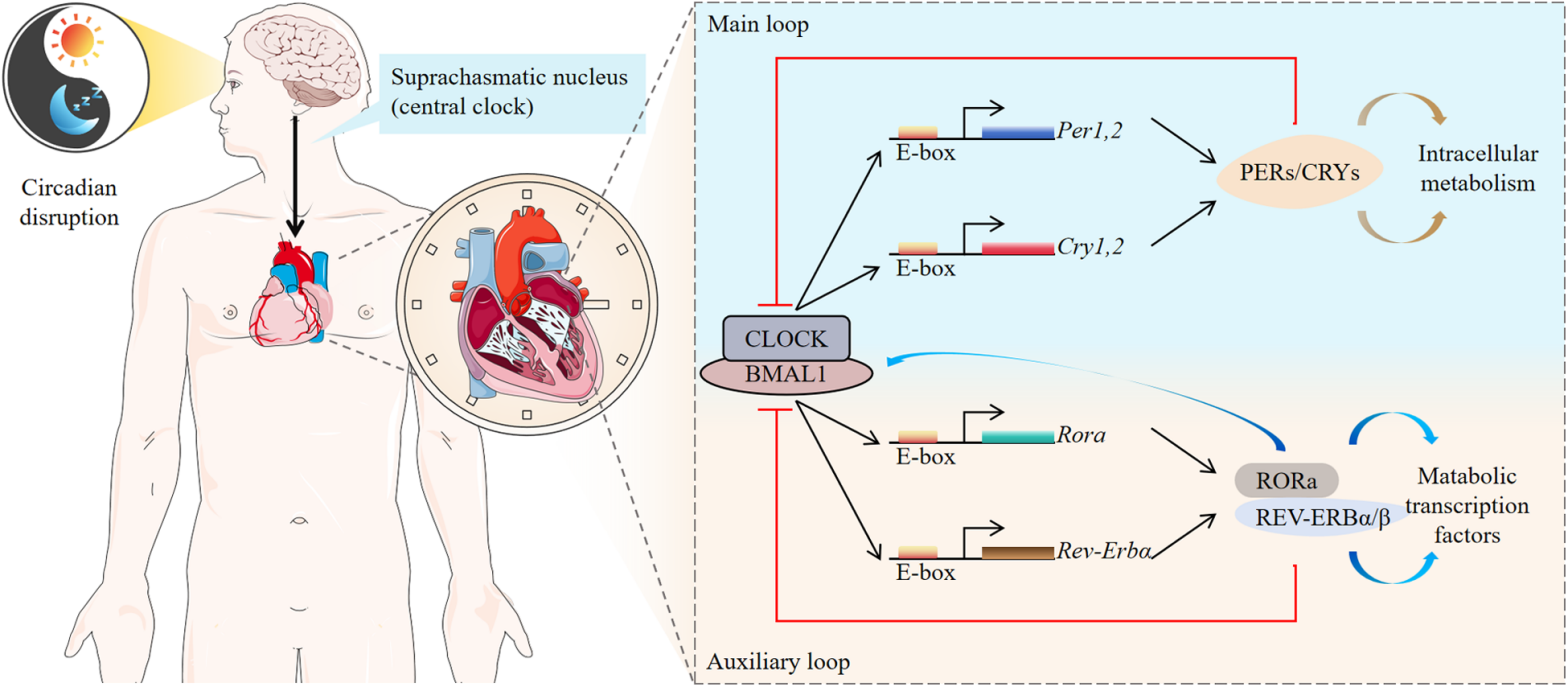
Molecular clocks in humans. The human molecular clock is driven by external cues, such as light and diet, which synchronize the central oscillator, or “master clock,” located in the suprachiasmatic nucleus (SCN). These cues reset peripheral oscillators found in nearly every cell of the body. The molecular clock mechanism consists of interconnected transcriptional feedback loops. Circadian clock-controlled genes (CCGs) are expressed under the regulation of these molecular clocks, driving physiological processes such as cardiac function throughout the day and night. REV-ERB and retinoid-related orphan receptor (ROR) stabilize and enhance the feedback loop mediated by BMAL1.

The REV-ERB gene is located on chromosome 17, and expresses in various cells throughout the human body, including those in the liver, heart, lung, adipose tissue, skeletal muscle, and brain. This gene is a critical regulator of metabolism, mitochondrial biogenesis, inflammatory responses, and fibrosis (35). The REV-ERBα protein, comprising 614 amino acids, consists of four distinct domains: A/B, C, D, and E, each with specialized functions. The A/B domain is a highly active nuclear receptor region capable of ligand-independent cis-activation, facilitating nucleic receptor interactions with other family members. The C domain, a conserved region, is responsible for DNA binding activity and determines the selection of chaperone nuclear receptors. The D domain, a flexible hinge region, contains nuclear localization signals. The E domain, characterized by proline- and acidic amino acid-rich regions (LBD), binds ligands, enabling dimerization and activation, thereby functioning as a transcription factor to regulate downstream target gene transcription. The LBD's primary structure includes ligand-binding pockets, especially the activation function 2 (AF-2) region, which features helices (H12), a co-regulatory factor-binding surface, and a dimerization surface. Notably, REV-ERBα lacks the AF-2 region, precluding transcriptional activation. Instead, REV-ERBα directly binds target gene promoters and enhancers, recruiting the nuclear receptor co-repressor (NCoR) and histone deacetylase 3 (HDAC3) complex to the REV-ERBα response element, thereby suppressing downstream gene transcription. Upon ligand binding, REV-ERBα undergoes a conformational change that enhances the recruitment of transcriptional co-regulatory proteins to receptor-specific gene promoter complexes, further inhibiting transcription (13). REV-ERBα also interacts with the retinoic acid-related orphan receptor α (RORα) to competitively regulate circadian rhythm oscillations (36). REV-ERBα inhibits the transcription and translation of the circadian clock components CLOCK and BMAL1 (also referred to as ARNTL), which form heterodimers that bind E-box elements to drive the transcription of core clock molecules and downstream targets (37). Conversely, RORα competes with REV-ERBα for ROR response element (RORE) binding, activating BMAL1 and CLOCK transcription (37). This interplay establishes a self-sustaining feedback loop involving REV-ERBα, ensuring robust and precise circadian clock regulation. Circadian rhythms orchestrate various physiological processes in a time-dependent manner to maintain homeostasis despite environmental factors, such as circadian circulation, food intake, and physical activity. Disruptions in circadian rhythm caused by gene mutations, shift work, exposure to artificial light, irregular eating patterns, or abnormal sleep cycles adversely affect human health (Figure 2).

### 3.2 Role of REV-ERB in autophagy, inflammation, immunity, metabolism, fibrosis, and ferroptosis

#### 3.2.1 REV-ERB and autophagy

Autophagy, a highly conserved intracellular degradation system, is essential for maintaining cellular homeostasis under stress conditions. Eukaryotic autophagy involves the use of lysosomes for the degradation of cytoplasmic proteins and damaged organelles of cells under the regulation of the autophagy-related gene (Atg) (38–40). NR1D1 is involved in the autophagy process occurring within various organelles, including mitochondria and lysosomes. Autophagy failure reportedly promoted cell degeneration, age-related senescence, tumor formation, and infection in mice and has also been speculated to play a key role in human diseases (41).

REV-ERBα regulates the oxidative function of skeletal muscle by regulating the genes involved in the different steps of mitophagy (42). SR9009 interacts with the core autophagy gene Atg5, inhibits autophagy activity, and plays an anti-tumor role in SCLC (small cell lung cancer) (43). In hepatic stellate cells (HSCs) (44), SR9009 regulates the signaling pathways associated with growth and survival, such as protein kinase B (AKT)/mammalian target of rapamycin (mTOR)/P70S6K (p70 ribosomal protein S6 kinase) pathway, also inhibits AV (autophagosome) biogenesis. The expression levels of REV-ERBα and REV-ERBβ in the granulosa cells of patients with polycystic ovary syndrome (PCOS) are lower than those in healthy people. According to *in vitro* studies, the overexpression of REV-ERBα and REV-ERBβ promotes the expression of mitochondrial biosynthetic genes and inhibits autophagy in granulosa cells. REV-ERBα and REV-ERBβ inhibit granulosa cell apoptosis and promote proliferation. In a mouse PCOS model (45), the REV-ERB agonist SR9009 reportedly improved abnormal follicular development by promoting mitochondrial biosynthesis and inhibiting autophagy. A study (46) reported that the negative regulation of REV-ERBα provided partial protection against glucose toxicity and cytokine-induced β-cell apoptosis. Sulli et al. (47) reported that the agonists of REV-ERBs (SR9009/SR9011) specifically targeted cancer cell *de novo* fatty acid synthesis and early autophagy-related pathways in A172 Glioblastoma (GBM) cells by sharply reducing the expression levels of the key rate-limiting enzymes involved in *de novo* lipogenesis. The agonists of REV-ERBs (SR9009/SR9011) exert a specific damage effect on cancer cells and oncogene-induced senescent cells but not on the viability of normal cells or tissues. According to a study (48), the agonist of REV-ERBs named GSK4112 could stimulate the autophagy flow of human macrophages and the expressions of key genes involved in lysosomal biogenesis through the activation of NR1D1 via the transcription factor EB (TFEB)-related pathway. A recent study (49) demonstrated that the transcription factors TFEB and Transcription factor binding to IGHM enhancer (TFE3), which are the major regulators of autophagy and lysosomal biogenesis, do not regulate NR1D1 expression by forming a complex with BMAL1-CLOCK and rather than directly binding to the NR1D1 promoter to drive the expression of REV-ERBα. Overexpression of TFEB and TFE3 in cells and knockdown of endogenous NR1D1 (50) leads to the expression of autophagy genes and enhancement of the autophagy flux. Therefore, the opposite, although interrelated roles of TFEB and TFE3 along with NR1D1, determine the time of autophagy activation.

#### 3.2.2 REV-ERB and inflammation

Circadian changes during inflammation and immune function have been observed in different physiological and pathological processes of humans and animals. However, the molecular mechanisms underlying these changes and the mediating cell types remain unknown. The biological clock controls several inflammatory processes, and the disturbed biological clock may lead to or aggravate inflammation. REV-ERB is vital in the inflammation process mediated by various cell types. REV-ERB regulates the inflammatory response by reducing the secretion of inflammatory cytokines, regulating gene transcription and the NOD-like receptor thermal protein domain associated protein 3 (NLRP3) inflammasome pathway, and inhibiting macrophage polarization.

The study revealed that aged mice exhibit time-dependent differences in controlling Streptococcus pneumoniae infection. Transcriptomic analysis of pulmonary circadian rhythms demonstrated significant alterations in the rhythmic expression of the core clock gene REV-ERBα and the lung's apelin/apelin receptor pathway. Further mechanistic investigations indicated that REV-ERBα mediates the host defense function of alveolar macrophages by regulating the apelinergic signaling axis. Pharmacological inhibition of REV-ERBα enhanced the resistance of aged mice to pneumococcal infection (51). In a study (52), the expression patterns of the biological clock-related genes in the thyroid of patients with autoimmune thyroiditis (AIT) were altered. Animal experiments have demonstrated that chronic circadian rhythm disorders cause significant oscillations in the biological clock-related genes, such as Bmal1, Clock, Per2, Cry1, Ror, and REV-ERB, in addition to causing increases in the secretion of tumor necrosis factor-α (TNF-α), interferon-γ (IFN-γ), and anti-thyroglobulin antibodies, thereby aggravating the inflammatory response of AIT. GSK4112 or SR9011 (NR1D1 agonist) attenuates microglia-mediated neuroinflammation by blocking the nuclear translocation of the NF-κB subunit p65 and inhibiting the expressions and secretion of pro-inflammatory cytokines [such as interleukin 6 (IL-6) and TNFα] in a dose-dependent manner (53). The pretreatment of primary chondrocytes with SR9009 (REV-ERB agonist) reportedly blocked the secretion of inflammatory molecules (matrix metalloproteinase, MMP3, MMP9, and MMP13) and cytokines (interleukin-1 β and tumor necrosis factor) in the cells induced by lipopolysaccharide. The repeated intra-articular treatment of SR9009 could prevent sodium iodoacetate-induced mechanical hypersensitivity and partially reduce knee joint injury in mice (54). A study of the peripheral blood mononuclear cells (PBMC) of young people after mild-to-moderate COVID-19 infection revealed that the expression of REV-ERBα in PBMC after COVID-19 infection was decreased upon LPS stimulation, indicating that REV-ERBα plays a role in both circadian rhythm control and inflammatory pathway (55). Studies have demonstrated that REV-ERBs inactivate the transcription of matrix metalloproteinase 9 (MMP9) and chemokine receptor (CX3CR1) in macrophages by inhibiting the transcription of enhancer-derived RNA (eRNA) (56). TH17 is a pro-inflammatory immune cell that prevents bacterial and fungal infections on the mucosal surface. REV-ERB α links the cellular development of TH17 to the circadian clock network, and its deletion increases the expressions of TH17-mediated pro-inflammatory cytokines and enhances the inflammatory response (57, 58). Dysregulation of the NLRP3 signaling cascade is associated with several inflammatory and metabolic diseases, including rheumatoid arthritis, gout, atherosclerosis, or type-2 diabetes. The biological clock controls several inflammatory processes, and the interrupted clock may lead to or aggravate inflammation. The circadian clock controls the expression and activation of NLRP3, thereby controlling the secretion of interleukin IL-1 β and IL-18 in different tissues and immune cells, particularly in macrophages. The circadian rhythm oscillation of the NLRP3 signal reportedly disappeared in the circadian clock disorder model, leading to the generation of peritonitis, hepatitis, or colitis (59). Morioka et al. demonstrated that REV-ERBα and REV-ERBβ mRNAs were expressed in cultured rat spinal cord microglia. Treatment of a culture of rat spinal microglia with SR9009 significantly blocked the lipopolysaccharide-induced increases in the expression levels of IL-1 β and IL-6 mRNA. Treatment of cultured rat spinal microglia with SR9009 significantly blocked lipopolysaccharide-induced increases in IL-1 β and IL-6 mRNA expression levels. Activation of REV-ERB exhibited analgesic effects in the spinal dorsal horn by negatively regulating spinal microglial activity (60). The findings demonstrate that ischemic stroke during the sleep phase (ZT06) significantly reduces the expression level of Rev-Erbα and exacerbates neuroinflammatory responses as well as stroke severity. Pharmacological intervention experiments reveal that the Rev-Erbα agonist SR9009, administered at ZT06, significantly alleviates neuroinflammation, reduces cerebral infarct volume, and downregulates the expression of the NLRP3 inflammasome in monocytes and neutrophils, as well as in brain tissue. Furthermore, SR9009 treatment markedly downregulates the expression of the pro-inflammatory cytokine TNFα while upregulating the expression of the anti-inflammatory cytokine IL-10. However, slight changes were found under the same intervention during the awake phase (ZT18) (61).

#### 3.2.3 REV-ERB and metabolism

Obesity and circadian rhythm disruption represent global health challenges, significantly elevating the risk of metabolic disorders. Adipose tissue and circadian rhythms are integral to regulating energy homeostasis, and their dysregulation is intricately associated with the pathogenesis of obesity. Circadian rhythm is closely related to metabolic regulation. The experimental study of the circadian rhythm gene expression profile revealed that approximately 3%–10% of the transcriptome is regulated by circadian rhythm, with the highest involvement in regulating basic metabolic processes (62). In the liver, the circadian rhythm regulates the basic metabolic processes, such as glycolysis, fatty acid metabolism, cholesterol biosynthesis, xenobiotics, and intermediate metabolism, which is realized through control of the rate-limiting steps of these processes. Studies in mice have demonstrated that genetic damage to the core clock gene leads to abnormal metabolic phenotypes, such as obesity, dyslipidemia, diabetes, hypertension, and HF (13). Circadian rhythm disorders due to shift work or lack of sleep are closely related to metabolic syndrome (63). Numerous studies have confirmed that ROR and REV-ERB coordinate the core circadian oscillators and clock-controlled genes to regulate metabolic pathways.

REV-ERB is expressed highly in the adipose tissue and exhibits a circadian rhythm, which is necessary for adipogenesis. A study (64) reported that REV-ERB inhibits adipogenesis by inhibiting the expression of peroxisome proliferator-activated receptor γ2, (PPARγ2), which is the primary transcriptional regulator of adipogenesis, and the dynamic change in the REV-ERB protein levels involved an initial increase followed by a decrease, leading to adipocyte differentiation (64). Studies have demonstrated that the double knockout of REV-ERBα/β caused a significant increase in the levels of TAG in the liver along with hepatic steatosis (65). An imbalance in the cholesterol metabolism plays a critical role in atherosclerosis. Cholesterol is a major part of the cell membrane and a significant metabolic precursor of the biosynthetic pathway, including the synthesis pathway for steroid hormones, vitamin D, and bile acids. The rate-limiting enzyme 3-hydroxy-3-methylglutaryl-coenzyme A reductase (HMGCR) and sterol regulatory element binding protein 2 (SREBP2) are the key regulators of cholesterol homeostasis (66). REV-ERBα participates in the circadian rhythm of sterol regulatory element binding protein (SREBP) activity regulation, and further the target genes related to cholesterol and lipid metabolism. REV-ERBα also participates in rhythmic bile acid metabolism regulation via regulating the expression of cholesterol 7 αhydroxylase (CYP7A1) (67). Studies have demonstrated that treatment with synthetic REV-ERB agonists decreases the plasma levels of cholesterol and the liver levels of the rate-limiting enzyme 3-hydroxy-3-methylglutaryl coenzyme a reductase in the biosynthesis reaction of cholesterol in mice. The REV-ERB agonist SR9009 reportedly reduced the plasma levels of cholesterol in wild-type mice and low-density lipoprotein receptor (LDLR)-deficient mice, while reducing the expressions of the related genes in the cholesterol biosynthesis pathway (66). REV-ERB ligands significantly regulate circadian behaviors and the rhythmic expression of core clock genes in the hypothalamus of mice. They also modulate the circadian expression patterns of metabolic genes in the liver, skeletal muscle, and adipose tissue, thereby promoting increased energy expenditure. Intervention with REV-ERB agonists in diet-induced obese mouse models effectively reduces fat mass and markedly improves dyslipidemia and hyperglycemia, thereby alleviating obesity (68).

REV-ERB agonists could effectively inhibit the expressions of fatty acid synthase (FASN), stearoyl-CoA desaturase 1 (SCD1), and PPAR-γ coactivator 1 β (PGC-1 β) in diet-induced obese mice, in addition to decreasing fat mass, improving dyslipidemia and hyperglycemia, and reducing obesity. Following eight weeks of continuous light exposure, mice exhibited increased body weight, insulin resistance, elevated white fat mass, and altered expression of circadian clock genes. Chronic administration of SR9009 restored the expression of REV-ERBα and REV-ERBβ in mice subjected to either a 24-h light cycle or constant light while suppressing the expression of Bmal1 in both white adipose tissue (WAT) and brown adipose tissue (BAT). By inhibiting adipogenesis, SR9009 effectively reduced weight gain, improved insulin resistance, and decreased white fat mass in mice under these lighting conditions (69). REV-ERBα (−/−) mice exhibited obesity and mild hyperglycemia under a regular diet. Moreover, the REV-ERBα (−/−) mice with overexpressed lipoprotein lipase (Lp1) gene under a high-fat diet exhibited promoted utilization of muscle fatty acids and fat overload (70). Heme reportedly inhibits hepatic gluconeogenic gene expression and glucose output via REV-ERBα-mediated genes (71). According to a study (72), the nuclear receptor REV-ERBs in GABAergic (γ-aminobutyric acid production) neurons (SCNGABA neurons) in the suprachiasmatic nucleus (SCN) control the rhythmic expressions of genes involved in neurotransmission within the SCN, regulate insulin secretion, and inhibit the circadian rhythm of hepatic glucose production in mice, while exerting no effects on the circadian rhythms of eating or regular light and dark cycle movement behavior. A clinical observational study demonstrated (73) that the novel pan-PPAR agonist Chiglitazar significantly reduces low-density lipoprotein cholesterol (LDL-C), free fatty acids (FFA), 3:00 a.m. blood glucose, and fasting blood glucose levels. Notably, its glucose-lowering effect is independent of lipid metabolism regulation mechanisms and may be modulated by the REV-ERB nuclear receptor pathway. This finding provides new theoretical evidence for the glucose-lowering mechanism of Chiglitazar, suggesting its potential role in glycemic regulation via targeting molecular pathways associated with circadian rhythms.

#### 3.2.4 REV-ERB and fibrosis

REV-ERB is primarily expressed in the liver, heart, lungs, adipose tissue, skeletal muscle, and brain, serving as a key regulator of fibrosis (35). Transforming growth factor-β (TGF-β), the central mediator of tissue fibrosis plays a critical role in the fibrotic response to injury. Chronic inflammation is increasingly recognized as a major contributor to morbidity and mortality (74). TGF-β has been shown to induce the expression of Bmal1 and Clock while suppressing PER1/2, REV-ERBα, and RORα, thereby influencing circadian clock genes (75).

In a study by Cunningham et al. (76), REV-ERBα inhibited differentiation and collagen secretion in cultured embryonic lung fibroblasts and pulmonary myofibroblasts derived from patients with pulmonary fibrosis. Conversely, in REV-ERBα-deficient (REV-ERB −/−) mice, pulmonary myofibroblasts exhibited increased collagen-1 and α-smooth muscle actin (α-SMA) secretion. The same study observed that the mRNA and protein levels of BMAL1, PER2, CRY1, and REV-ERBα were reduced in the peripheral blood mononuclear cells (PBMCs), lung tissue, and sputum cells of smokers and patients with chronic obstructive pulmonary disease (COPD) compared to non-smokers (77). Studies have demonstrated that REV-ERBα agonists, such as SR9009 and GSK4112 inhibit fibroblast differentiation in human fetal lung fibroblasts (HFL-1) induced by TGF-β or cigarette smoke (CS) (78). These agonists also attenuate the inflammatory response and pulmonary fibrosis in airway epithelial cells and mouse lung fibroblasts exposed to lipopolysaccharide (LPS) or cigarette smoke (79).

Hepatic stellate cells (HSCs), mesenchymal cells located in the space of Disse between hepatocyte basements and sinusoidal endothelial cells, play a vital role in liver fibrosis (80). In their resting state, HSCs primarily store vitamin A and retinol. However, liver damage activates HSCs, transforming them into myofibroblasts with contractile, migratory, and fibrogenic properties. Chronic liver injury leads to repeated HSC activation, resulting in cirrhosis, hepatocellular carcinoma (HCC), and fibrous tissue accumulation that disrupts liver structure, increases portal vein pressure, and impairs liver function. Research by Li et al. (81) indicated that REV-ERBα protein expression was upregulated in activated HSCs and damaged liver tissue. The REV-ERB ligand SR6452 improved liver fibrosis and portal hypertension (PH) in rats by inhibiting cytoplasmic REV-ERBα expression, reducing α-SMA and TGF-β levels, and suppressing HSC activation. In a mouse model of non-alcoholic steatohepatitis (NASH), SR9009 was reported to reduce glucose levels, improve glucose tolerance, and inhibit the expression of fibrotic markers, such as collagen α1(III) (COLl3A1), α-SMA, STAT1, MMP13, TIMP1, and TGF-β. These effects reduced liver fibrosis and inflammation and improved liver function (82). In another study using a CCl4-induced liver fibrosis mouse model, the REV-ERB agonist SR9009 inhibited autophagosome biogenesis, fibrosis-related gene expression, and HSC proliferation through the regulation of P70S6K (44). These findings underscore the potential therapeutic value of REV-ERBα agonists in fibrosis and associated pathologies treatments.

#### 3.2.5 REV-ERB and mitochondrial function

Mitochondrial biosynthesis is a complex process involving the growth and division of mitochondria to meet increased energy demands (83). This process relies on coordinated signaling pathways to ensure the production of new mitochondria. Studies have established that REV-ERBs are essential in mitochondrial biogenesis (84). For instance, Woldt et al. (42). demonstrated that REV-ERBα knockout in skeletal muscle inactivated the Lkb1-Ampk-Sirt1-Ppargc-1α signaling pathway. This inactivation reduced mitochondrial content, impaired mitochondrial oxidative function, increased autophagy, and diminished exercise capacity collectively. In contrast, overexpression of REV-ERBα increased mitochondrial numbers and improved mitochondrial respiration. In patients with polycystic ovary syndrome (PCOS), the expression levels of REV-ERBα and REV-ERBβ in ovarian granulosa cells were significantly lower than in healthy controls (45). *In vitro* experiments with human ovarian granulosa cells (KGN) showed that overexpression of REV-ERBα and REV-ERBβ upregulated the mitochondrial biosynthesis genes, such as PGC-1α, NRF1, and TFAM, while reducing mitophagy. Furthermore, treatment with the REV-ERB agonist SR9009 promoted mitochondrial biosynthesis, alleviating follicular dysplasia. Mitochondrial dynamics involves the processes, such as fission, fusion, and subcellular translocation, which are crucial for maintaining mitochondrial DNA integrity and respiratory function (85). In many cell types, mitochondria exist as dynamic networks, with fission and fusion regulating their function and adaptation. In a mouse model of Parkinson's disease (PD) induced by the neurotoxin 1-methyl-4-phenyl-1,2,3,6-tetrahydropyridine (MPTP) and in SH-SY5Y neuronal cells, sinapic acid was observed to exert protective effects against MPTP-induced PD. This protection involved increased expression of REV-ERBα protein and decreased levels of mitochondrial fission proteins, including dynein-related protein 1 (DRP1) and phosphorylated DRP1 at SER616 (86). These findings emphasize the essential roles of REV-ERBs in mitochondrial biosynthesis and dynamics, with significant implications for understanding and managing metabolic and neurodegenerative disorders.

#### 3.2.6 REV-ERB and ferroptosis

**Figure 3:**
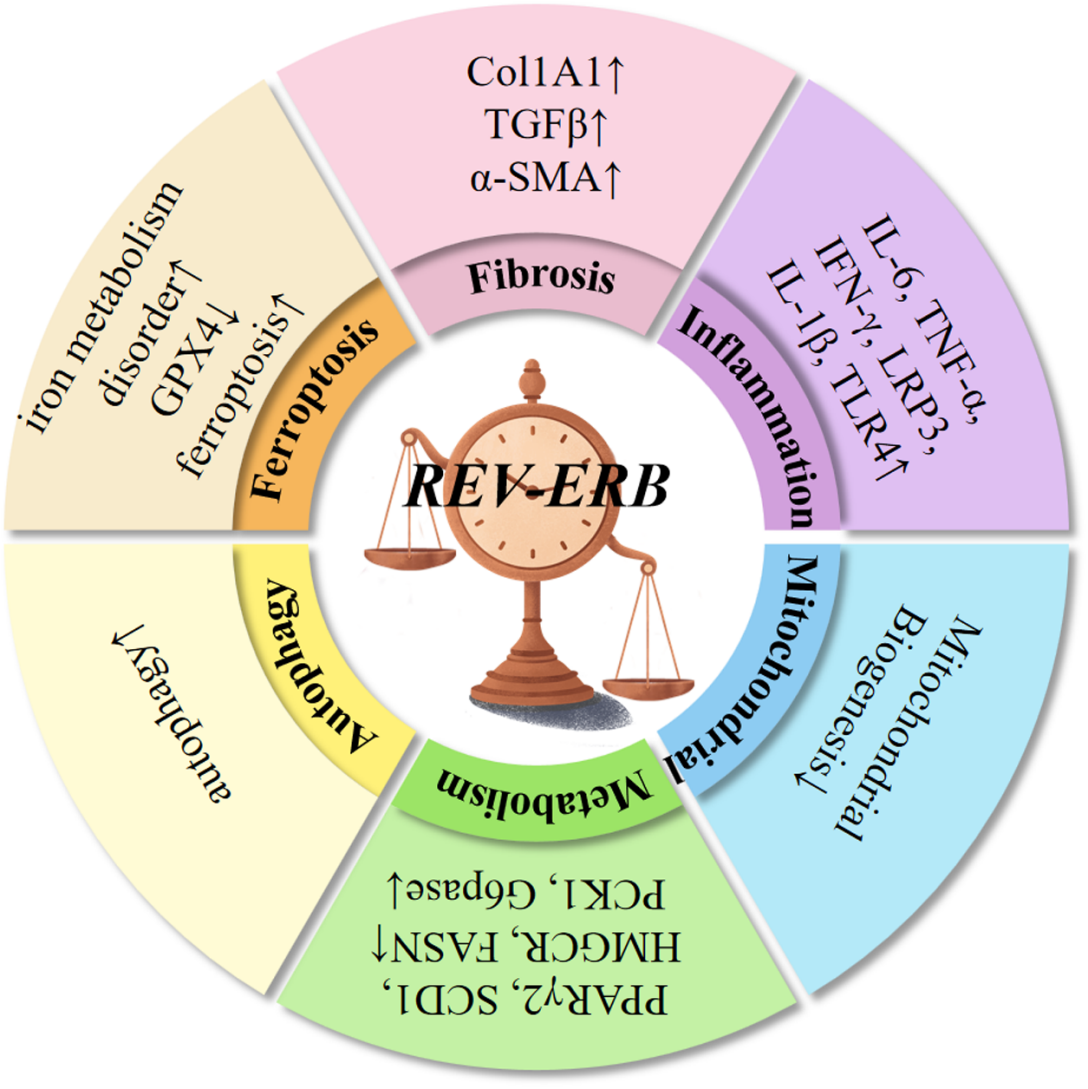
REV-ERBα/β proteins exhibit robust 24-h oscillations and regulate various physiological functions. These proteins inhibit autophagy, suppress gluconeogenesis, regulate insulin and glucagon levels, and maintain rhythmic glucose oscillations. In the liver, REV-ERBα/β regulate lipid synthesis, transport, bile acid metabolism, and control adipocyte differentiation and adipose tissue expansion. They also contribute to fatty acid oxidation in skeletal muscle and the heart. As inflammatory regulators, REV-ERBα/β are involved in NF-κB signaling, NLRP3 inflammasome activation, transcription of inflammation-related genes, macrophage polarization, and immune cell development. Additionally, REV-ERBα/β inhibit fibrosis across multiple organs, regulate mitochondrial biogenesis, prevent intracellular iron metabolism disorders, and suppress ferroptosis.

Ferroptosis is an iron-dependent, non-apoptotic form of cell death, characterized by increased lipid peroxidation (87). Type 2 diabetes was induced by a high-fat diet (HFD) and an intraperitoneal injection of streptozotocin (STZ) in mice with cardiac-specific knockout of the REV-ERBα gene (88). Meanwhile, high glucose (HG) levels and high palmitic acid (PA) levels were administered to induce glycolipid toxicity in H9C2 cardiomyocytes *in vitro*. It was observed that after REV-ERBα was knocked out, the disorder of iron metabolism in the myocardium was aggravated, the expression of glutathione peroxidase 4 (GPX4) was decreased, the cardiac function significantly deteriorated in the diabetic mice, and the indicators of myocardial inflammation, myocardial fibrosis, and oxidative stress were significantly increased. Contrary to the view that REV-ERBα/β deficiency is associated with poor health conditions, such as cancer, metabolic disorders, and severe inflammation, a study (89) demonstrated that REV-ERB deficiency inhibits ferroptosis and improves folic acid-induced acute kidney injury. Another study (90) on renal injury induced by aristolochic acid I (AAI, a typical AA) reported that the kidney-specific knockout of REV-ERBα in mice decreased the sensitivity of these mice to AAI-induced ferroptosis and renal injury. Meanwhile, treatment with siRNA or SR8278 (a REV-ERBα antagonist) *in vitro* reduced the aristolochic lactam I (ALI)-induced ferroptosis in mouse renal tubular epithelial cells (mRTECs). In addition, the REV-ERBα antagonistic effect of SR8278 alleviated the AAI-induced ferroptosis and renal injury in mice. ArBu exhibits a broad spectrum of anti-tumor activity (91). Treating human gastric cancer cells with ArBu increases the expression of REV-ERBα and causes ferroptosis. Numerous studies have demonstrated that REV-ERB inhibition could be harmful or beneficial, depending on the type of disease (Figure 3).

## 4 Role of REV-ERB in cardiovascular diseases

### 4.1 REV-ERB and atherosclerosis

CVDs present high morbidity and mortality and are associated with tremendous healthcare costs that are ever-increasing. According to the 2019 Global Burden of Disease (GBD) study, the total number of cases of cardiovascular diseases increased from 271 million in 1990 to 523 million in 2019. The number of deaths due to CVDs has been on a steady rise, from 12.1 million deaths reported in the year 1990 to 18.6 million deaths reported in 2019, with the latter accounting for 33% of all deaths worldwide (92). Atherosclerosis is a vital risk factor for CVDs. Its pathogenesis is characterized by cholesterol deposition, smooth muscle hyperplasia, inflammatory cell infiltration, and connective tissue accumulation, manifesting as plaque formation (atherosclerosis) in the intima of the arterial wall. Changes in the expressions of biological clock-related genes contribute to the pathogenesis of atherosclerosis. Aortic aneurysm, rupture, and dissection are among the most life-threatening arterial diseases and are closely associated with atherosclerosis. Plaque deposition, inflammation, and arterial wall remodeling driven by atherosclerosis contribute to the weakening of vessel walls, increasing the risk of these conditions. Studies have demonstrated that the circadian rhythms influencing blood pressure, heart rate, vascular tone, and vasoconstrictor hormone levels also affect the incidence of aortic aneurysms and dissections. The early morning hours are particularly critical, as physiological processes, such as plaque rupture, hypercoagulability, and coronary vasoconstriction are often aggravated during this time, heightening the risk of cardiovascular events (93, 94). In contrast to ischemic events, which are primarily influenced by acute occlusion of coronary arteries, aortic diseases emerge as chronic outcomes of arterial wall deterioration linked to prolonged exposure to atherosclerotic factors. While circadian rhythm disorders exacerbate the risk, these conditions should be distinctly categorized as arterial diseases mediated by structural and inflammatory mechanisms rather than I/R injury (95).

The severity of atherosclerotic lesions is determined by the balance of monocyte recruitment, macrophage excretion, proliferation, survival, and arterial wall apoptosis (96). Apolipoprotein CIII (apoCIII) functions critically in plasma triglycerides and residual lipoproteins. The presence of triglyceride-rich residual lipoproteins in plasma is closely related to atherosclerosis. In the human liver HepG2 cells expressing apoCIII, silencing the REV-ERBα gene could specifically inhibit the apoCIII gene promoter activity (97). A key step before the formation of atherosclerotic lesions is the migration of monocytes to the arterial wall and their differentiation into macrophages (98). Macrophages are the primary organizers of plaque inflammation (98, 99). Sato et al. reported that REV-ERBα inhibits the expression of the IL-6 gene in macrophages, either directly or indirectly, via RORE and nuclear factor-κB (NF-κB) response elements in the IL-6 promoter region, and the expression of the IL-6 gene was increased in the peritoneal macrophages of the mice lacking REV-ERBα (100). Several NRs are associated with developing CVDs and atherosclerosis (101). A study demonstrated that shRNA-mediated REV-ERBα deficiency, especially in hematopoietic cells, exacerbated the development of atherosclerotic lesions in LDLR (−/−) mice. At the cellular level, REV-ERBα knockdown in bone marrow-derived monocytes promoted the shift of pro-inflammatory macrophages to the anti-inflammatory phenotype (102). In the same study, treating LDL receptor-deficient mice with the REV-ERBα agonist SR9009 reduced the polarization of mouse macrophages (BMDMs) to pro-inflammatory M1 macrophages which increased the polarization of BMDMs to anti-inflammatory M2 macrophages. After seven weeks of this treatment, the size of the atherosclerotic plaques in the treated mice was significantly reduced (103). In another study (104), REV-ERBα expression in primary human macrophages could be induced using the synthetic ligands of the liver X receptor (LXR). Overexpression of REV-ERBα inhibited the induction of toll-like receptor TLR-4 by LXR agonists. However, its knockdown increased the expression of TLR-4, indicating that this was the molecular mechanism underlying the regulatory effect of REV-ERBα on cholesterol homeostasis. Vulnerable plaque rupture is the main trigger for most acute cardiovascular events. The study of vulnerable plaque models in hypercholesterolemia ApoE (−/−) mice and NR1D1 (−/−) ApoE (−/−) mice revealed that NR1D1 deficiency could significantly increase the vulnerability/rupture of plaques (105). The incidence of intraplaque hemorrhage and spontaneous plaque rupture with intravascular thrombosis, together with the findings of *in vitro* experiments conducted using mouse bone marrow-derived macrophages (BMDMs), confirmed that NR1D1 plays a protective role by inhibiting macrophage pyroptosis. Table 1 summarizes studies involving REV-ERB in CVDs, detailing the type of CVDs, the animal/cell model used, and pathophysiological implications.

**Table 1:** Summarizing studies involving REV-ERB in cardiovascular diseases.

Authors	Type of CVD	Animal/cell model	Pathophysiological implications	Other relevant factors
Luo et al. (111)	Cardiac fibrosis	Murine embryonic fibroblasts and cardiac fibroblasts with REV-ERBα/β deletion	REV-ERBα/β deletion in cardiac fibroblasts led to reduced viability and proliferation, increased migration, and myofibroblast activation, indicating its essential role in maintaining fibroblast homeostasis	SR9009, a REV-ERB agonist, suppressed cardiac fibroblast activation, but this effect was found to be REV-ERB-independent, highlighting the need for novel agonists
Zou et al. (125)	Doxorubicin-induced cardiotoxicity	*in vivo* mouse model and *in vitro* H9c2 cardiomyoblast cells	Activation of Rev-erbα by SR9009 decreased doxorubicin-induced apoptosis and oxidative stress, suggesting a protective role against cardiotoxicity. This effect is associated with the preservation of mitochondrial function and activation of the PGC-1α signaling pathway	The study suggests that Rev-erbα activation could serve as a cardioprotective strategy against chemotherapy-induced cardiotoxicity
Li et al. (126)	Heart failure due to pressure overload	Cardiomyocyte-specific REV-ERBα/β double knockout (cDKO) mice subjected to transverse aortic constriction (TAC)	cDKO mice exhibited severe cardiac dysfunction and ventricular dilation after pressure overload, indicating that REV-ERB is essential for cardiac stress response and remodeling. However, treatment with SR9009 improved heart function after pressure overload, suggesting that its beneficial effects might be independent of cardiac REV-ERB.	The study indicates potential off-target effects of SR9009 and suggests the need for more specific REV-ERB agonists to fully understand its role in cardiac function
Zhang et al. (112)	Heart failure	Mouse models of heart failure induced by transverse aortic constriction (TAC)	Pharmacological activation of REV-ERBα by SR9009 suppressed aberrant pathological gene expression, prevented cardiomyocyte hypertrophy, reduced fibrosis, and halted progression of advanced heart failure.	The study suggests that targeting REV-ERBα could be a novel therapeutic approach for heart failure by modulating gene networks involved in pathological remodeling
Zhang et al. (112)	Cardiac hypertrophy	Mouse models subjected to pressure overload and neonatal rat ventricular myocytes (NRVMs)	REV-ERBα activation by SR9009 prevented the development of cardiac hypertrophy and reduced fibrosis. In NRVMs, SR9009 treatment enriched metabolic pathways downregulated in heart failure, particularly fatty acid metabolism.	The study highlights the potential of REV-ERBα activation in preventing pathological cardiac remodeling and suggests metabolic modulation as a mechanism

### 4.2 REV-ERB and myocardial fibrosis

The prevalence of heart disease is increasing with time, with approximately 64.3 million cases of HF reported worldwide (106). Heart disease is associated with severe morbidity and mortality, along with a poor quality of life (107). The prevalence of all types of HF in patients aged 65 and above is approximately 11.8% in developed nations (108). Pathologically, HF is characterized by interstitial fibrosis, ventricular remodeling, and decreased ventricular compliance (109). In the heart of adult mammals, cardiomyocytes account for approximately 75% of the myocardial volume and are organized into 2–5 cell-thick layers. These myocardial cell layers are surrounded by interstitial extracellular matrix (ECM), which is mainly composed of fibrous collagen (110). As a mechanical scaffold, the epicardium is vital for contractile force transmission. In addition to type I collagen (the most abundant protein in the cardiac ECM) and type III collagen, the cardiac ECM consists of various glycoproteins, glycosaminoglycans, and proteoglycans (111). Moreover, it has a reservoir of stored potential growth factors and proteases, which are rapidly activated upon injury to stimulate repair. Several epidemiological studies have demonstrated that shift workers are at an increased risk of developing CVDs (112–115). Various non-standard schedules required by shift workers force sudden changes in their sleep time and light and dark exposure periods. These changes lead to a disordered endogenous circadian rhythm system, and disbalance the external body environment. The circadian rhythm system disorder caused by night shift work, besides causing an imbalance between the circadian rhythm system and the external light-dark cycle, also leads to an internal asynchrony among the different levels of the circadian rhythm system (116). Genome-wide gene expression analyses have revealed that shift work reprogrammed the cardiac circulatory transcriptome and led to cardiac fibrosis. REV-ERBα/β double deletion decreased the viability and proliferation of cardiac fibroblasts while increasing the migration of cardiac fibroblasts and myofibroblast activation. REV-ERBα/β are reported to maintain cardiac fibroblasts in a healthy resting state (117). The REV-ERB agonist SR9009 selectively inhibits abnormal pathological gene expression and prevents cardiomyocyte hypertrophy (118). The activation of REV-ERBα prevented the development of cardiac hypertrophy in mouse models, reduced the degree of fibrosis, and delayed the progression to advanced HF.

### 4.3 REV-ERB and myocardial ischemia

Early studies conducted on humans have revealed circadian rhythm patterns in the onset of acute myocardial ischemia (MI). The incidence of MI is higher in the morning than in the evening, aligning with the early-morning onset of other adverse cardiovascular events, such as unstable angina pectoris, sudden death, stroke, ventricular arrhythmia, cardiogenic shock, stent thrombosis, and transient MI. When the plaque narrows the arterial lumen, blood flow is limited, leading to ischemia of the distal tissue. When the lumen is completely blocked, blood flow is interrupted further, which may lead to non-fatal or fatal coronary artery disease or cerebrovascular events. Changes in the expressions of biological clock-related genes contribute to the pathogenesis of ischemic events. Studies have reported that in the morning, several physiological processes that might lead to plaque rupture, hypercoagulability, or coronary vasoconstriction are aggravated (94). Clinical studies have shown that the severity of MI is also heightened in the morning. Furthermore, the incidence of ischemic heart disease is significantly higher among shift workers compared to daytime workers, independent of lifestyle factors, such as smoking or age. Maintaining normal circadian rhythms or healthy sleep patterns is thus crucial for cardiovascular health (119).

Ischemic/reperfusion (I/R) injury studies in animal models provide insight into the circadian regulation of cardiac damage (120). For instance, rats subjected to left coronary artery occlusion followed by reperfusion showed altered circadian gene expression in ischemic regions (121). These changes included diminished peak expressions of critical clock genes, such as Npas2, Per1-3, Cry1-2, and REV-ERBα, and reduced amplitude of Bmal1, Clock, and other circadian regulators (122). This rapid inactivation of the biological clock in ischemic heart regions disrupts synchronization with the environment and neighboring heart regions (123).

Time-of-day studies in a mouse model of left anterior descending coronary artery occlusion demonstrated that ischemia during the sleep-to-awake transition (ZT12) resulted in larger infarct size, increased fibrosis, and impaired remodeling compared to ischemia during the awake-to-sleep transition (ZT0). Zhao et al. observed that shift workers with circadian rhythm disruption exhibited larger infarct sizes (5), reduced left ventricular ejection fraction (LVEF), and a higher risk of major adverse cardiac events. Mechanistic studies identified reduced Nr1d1 expression as a key driver of exacerbated MI outcomes in human and animal models. Cardiomyocyte-specific NR1D1 knockout mice showed increased infarct size, cardiomyocyte death, and worsened LVEF after MI and reperfusion injury. Therapeutic interventions targeting REV-ERB, such as intraperitoneal injections of the REV-ERB agonist SR9009, demonstrated significant benefits. These included improved survival rates, enhanced left ventricular function, reduced brain natriuretic peptide (BNP) levels, and lower inflammatory markers (e.g*.*, IL-6, Mcp1, Ly6g). SR9009 also mitigated the activation of NF-κB and MAPK signaling pathways (124). Furthermore, SR9009 modulated cardiac fibroblast function, weakening the NLRP3 inflammasome, reducing immune cell recruitment, and accelerating myocardial repair (125).

### 4.4 REV-ERB and heart failure

HF is characterized by many clinical symptoms resulting from various pathogenic factors impairing the heart structure and/or function. It has become one of the most serious threats to human health. Notably, approximately 13% of cardiac genes and nearly 8% of related proteins exhibit rhythmic patterns, and disruptions in these rhythms can contribute to the development of HF. To further investigate circadian rhythm-related genes that may aid in identifying and treating HF, a study (126) analyzed expression data from ischemic and dilated cardiomyopathy patients with or without HF, using datasets from the GEO database. The study identified 723 differentially expressed circadian rhythm-related genes (DEGs) in HF patients compared to healthy controls. Among these, the relative mRNA expression levels of CRY2 and BHLHE41 were significantly elevated in the HF group, while *ARNTL* and *NPAS2* expression levels were reduced. The cardiomyocyte circadian clock is crucial in myocardial systolic regulation, metabolism, and gene expression (34). Studies on Bmal1 (−/−) mice have demonstrated that Bmal1 deficiency leads to progressive myocardial pathological changes, first appearing at 36 weeks of age (24). These changes initially manifest as a transient increase in myocardial mass, followed by gradual ventricular dilation, ultimately resulting in HF and death. An *in vitro* working heart perfusion test revealed that systolic ventricular dysfunction coincided with the onset of dilation and failure. This was accompanied by the downregulation of two myosin heavy chain subtype mRNAs, sarcomeric structural abnormalities, and a shift in annexin isomer composition toward the stiffer N2B isomer.

The nuclear receptors REV-ERBα/β, critical to the circadian clock, have emerged as promising pharmacological targets for cardiac diseases. Pieterjan Dierickx et al. discovered that mice with cardiomyocyte-specific deletion of Rev-erb exhibit premature death due to dilated cardiomyopathy. Further mechanistic studies revealed that the absence of REV-ERB downregulates the expression of fatty acid oxidation genes through its direct target, E4BP4, and disrupts NAD+ biosynthesis, impairing cardiac metabolism and function (127). A study (128) using a mouse model of myocardial cell REV-ERB gene knockout (KO) compared the cardiac function of KO and WT mice. KO mice exhibited impaired systolic function, left ventricular enlargement, and a decreased ejection fraction starting at 4.5 months. By 6–8 months, most KO mice succumbed to cardiac dysfunction. Mechanistic studies revealed that heart REV-ERB plays a crucial role in enhancing the expression of fatty acid metabolism-related genes (FAO genes) during the light cycle while counteracting diet-induced activation of glucose metabolism genes in the dark cycle. REV-ERB knockout impaired oxidative lipid metabolism, leading to systolic and diastolic dysfunction. Fat reduction may serve as a predictor of adverse outcomes in advanced heart failure (HF), with circadian clock disruption identified as a key driver of lipid metabolism disorders. To further investigate the role of circadian rhythm disruption in lipid metabolism disorders associated with HF, a study (129) established an HF model and divided it into different groups: normal rhythm (LD), inverted rhythm (DL), a lentiviral vector carrying Bmal1 short hairpin RNA (LV-Bmal1 shRNA), and empty lentiviral vector control (LV-Control shRNA). Monitoring lipid metabolism levels revealed that BMAL1 protein levels in the adipose tissue of the LD group were lower than in the control group. In the normal-rhythm HF model, REV-ERBα protein levels increased, indicating circadian rhythm disruption. Additionally, HF rats exhibited decreased fat mass, increased ectopic lipid deposition, smaller adipocytes with lower lipid content, and fibrotic adipose tissue. Mechanistic studies further demonstrated that the disruption of the BMAL1/REV-ERBα circadian rhythm loop contributed to increased fat consumption in HF. Treatment with the REV-ERB agonist SR9009 reduced cardiac remodeling by decreasing AKT activity in aged WT mice (130). MEF2a and MEF2c are key regulators of the cardiac hypertrophy gene program. Studies have shown that ectopic expression of MEF2a or MEF2c in the heart can lead to dilated cardiomyopathy and HF (131, 132). A study (118) found that REV-ERBα, upon co-localization with MEF2a and MEF2c, could specifically inhibit MEF2a/MEF2c-driven cardiac hypertrophy and the aberrant activation of gene programs in HF, thereby playing a critical role in preventing ventricular remodeling.

**Figure 4:**
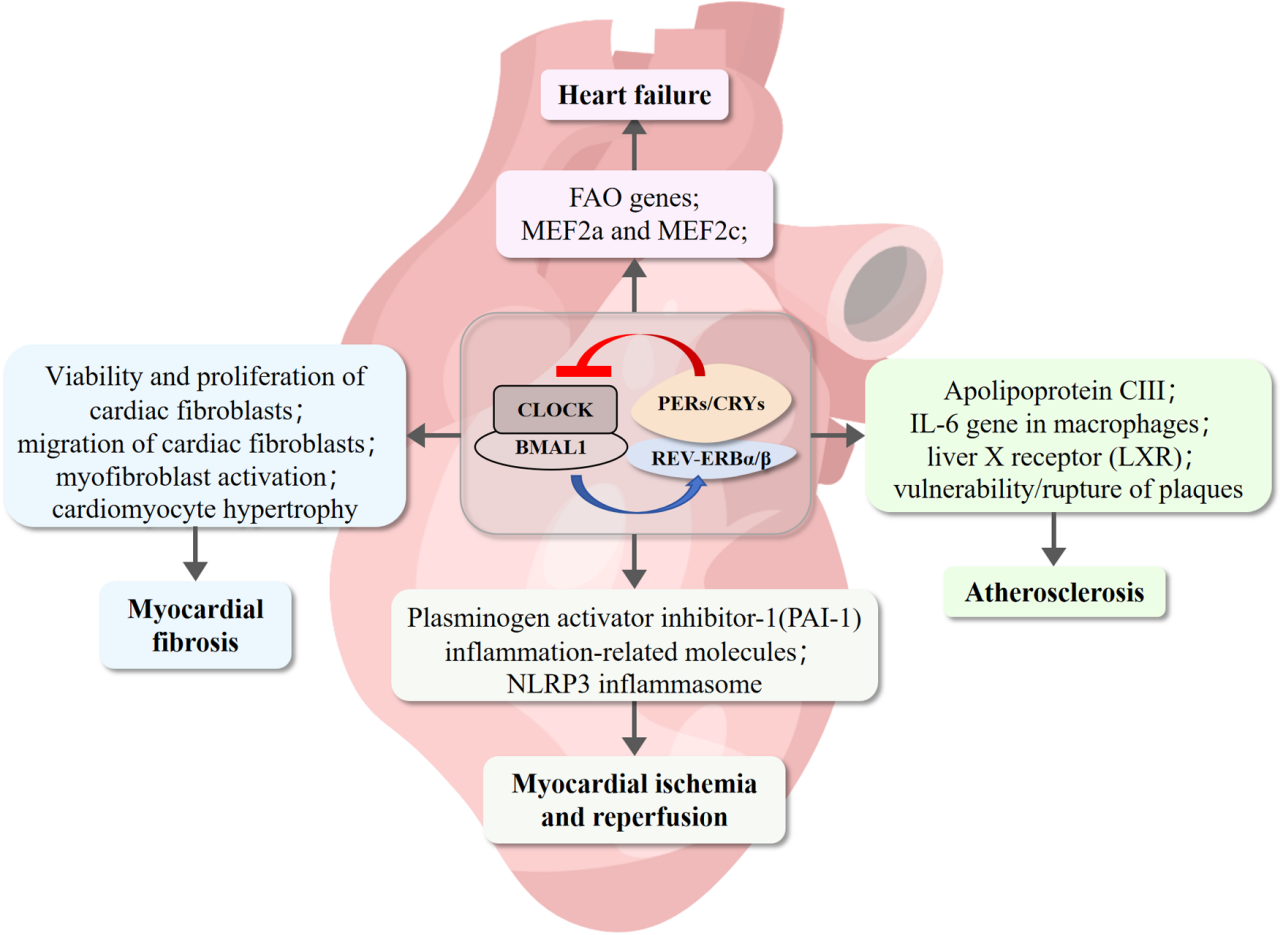
REV-ERB involvement of cardiovascular diseases pathogenesis. REV-ERB is implicated in the pathogenesis of cardiovascular diseases. Deficiency in REV-ERB increases the vulnerability and rupture of plaques. Its expression is significantly reduced in myocardial infarction and reperfusion injury. REV-ERB plays a crucial role in maintaining cardiac fibroblasts in a healthy resting state, preventing pathological gene expression, and inhibiting cardiomyocyte hypertrophy.

The pathological remodeling of the myocardium is associated with typical gene expression programs and had long been considered the result of the disease, until recently, when it was indicated to be the driver of the disease. Numerous studies have demonstrated that the pharmacological activation of REV-ERB selectively inhibits abnormal pathological gene expression and prevents cardiomyocyte hypertrophy (Figure 4). Therefore, regulating the gene network by targeting REV-ERBα could be a novel strategy for curing HF (118).

## 5 Conclusions

Circadian rhythms, intrinsic 24-h cycles regulating human physiology, play a fundamental role in maintaining health. Disruptions caused by modern lifestyles, work schedules, and increased screen time have led to widespread chronodisruption, contributing to adverse effects on sleep, physical health, and mental well-being. The regulation of circadian rhythms is crucial for cardiovascular health. Molecular mechanisms involving REV-ERB, a key circadian regulator, have shown protective roles against CVDs, such as atherosclerosis, MI/reperfusion injury, and HF. These findings highlight the importance of maintaining circadian rhythm stability to prevent and manage CVDs. Future research should continue to explore the interplay between circadian rhythm regulators and pathophysiological processes, aiming to develop effective therapeutic strategies for combating circadian rhythm-related disorders and enhancing overall health.
